# Editorial: Pathological reactions of cytotoxic lymphoid cells as universal therapeutic targets in cancer and autoimmune disease

**DOI:** 10.3389/fmed.2023.1186318

**Published:** 2023-04-25

**Authors:** Vadim V. Sumbayev, Bernhard F. Gibbs, Elizaveta Fasler-Kan

**Affiliations:** ^1^Medway School of Pharmacy, Universities of Kent and Greenwich, Chatham Maritime, United Kingdom; ^2^Department of Human Medicine, University of Oldenburg, Oldenburg, Germany; ^3^Department of Pediatric Surgery, Children's Hospital, Inselspital Bern, University of Bern, Bern, Switzerland; ^4^Department of Biomedical Research, University of Bern, Bern, Switzerland

**Keywords:** immune evasion, cancer, autoimmune disease, T cells, immune checkpoints

Malignant transformation of human cells is associated with the activation of immunosuppressive biochemical pathways. Cytotoxic lymphoid cells, mainly T lymphocytes and natural killer (NK) cells are capable of attacking and eliminating malignant cells. However, cancer cells operate effective immune evasion machinery, which includes immune checkpoint proteins and small molecular weight compounds as well as biochemical pathways responsible for their expression, production and secretion. These immune evasion networks create an immunosuppressive milieu and allow cancer cells to escape immune attack, thus leading to disease progression (1).

Immune evasion networks include programmed cell death protein (PD-1)/ programmed death ligand (PD-L1), Tim-3 (T cell immunoglobulin and mucin domain-containing protein 3)/galectin-9, V domain Ig-containing suppressor of T cell activation (VISTA), T-cell immunoreceptor with Ig and ITIM domain (TIGIT), for example, as well as signaling cascades involving transforming growth factor beta type 1 (TGF-β)-Smad-3 and interferon beta or gamma (IFN-β or IFN-γ) pathways (2–6). However, recent evidence demonstrated that certain small molecular weight compounds are also effectively involved in suppression of T cell function and thus evasion of anti-cancer immunity. Recently, concern has been expressed regarding the role of L-kynurenine (LKU) and its metabolites in suppression of cytotoxic lymphoid cell functions during cancer progression. LKU is an amino acid which is formed during L-tryptophan (L-Trp) catabolism in kynurenine pathway (7). A number of malignant tumors express indoleamine 2,3-dioxygenase 1 (IDO1) which supports LKU production by tumor cells (7).

These immune checkpoint proteins and pathways cooperate, thus potentiating their immune evasion effects. For example, galectin-9 induces granzyme B activation in cytotoxic T cells (5, 8) and cooperates with other immune checkpoint proteins, particularly VISTA or PD-L1, which block their intracellular anti-apoptotic machinery, resulting in programmed death of these T cells. Additionally, VISTA, Tim-3 and PD-L1 block the production of IL-2 by helper T cells thus preventing the activation of cytotoxic T cells (3, 8, 9). The TGF-β/Smad3 pathway can, on the one hand, upregulate expression of immune checkpoint proteins and, on the other hand, induce the differentiation of naïve T cells into Tregs which downregulate T cell-mediated attack of tumor cells. Examples of the various cross-links between immune checkpoint and immune evasion pathways are summarized in Figure 1.

**Figure 1:**
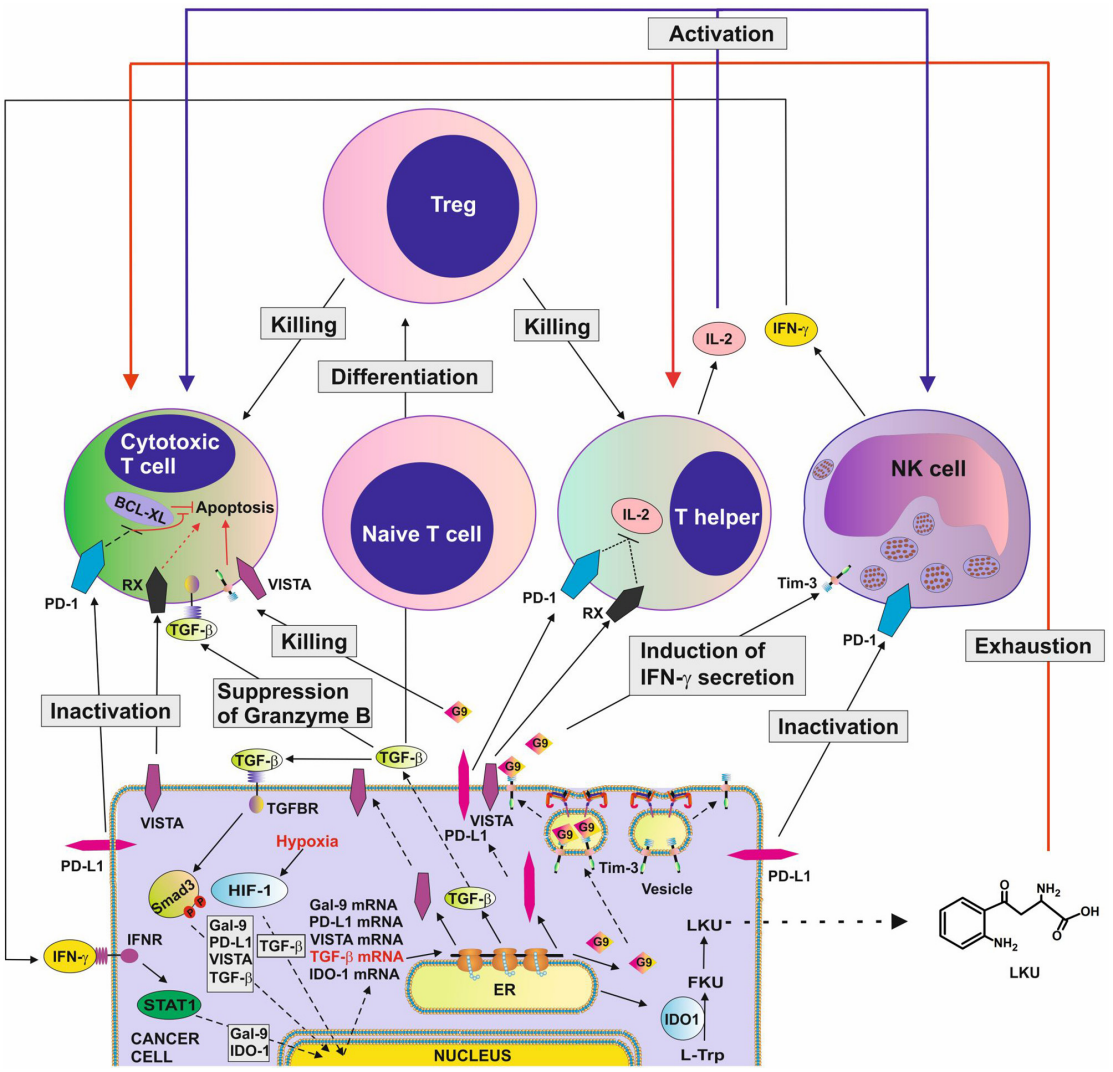
Complex immune evasion machinery operated by cancer cells. This scheme highlights how several immune evasion pathways could cross-link to potentiate anti-cancer immune evasion. The scheme is based on recent discoveries in the field and shows the complexity of the process. G9, galectin-9; RX, receptor for VISTA which remains to be identified; TGFBR ,receptor(s) for TGF-β; HIF-1, hypoxia-inducible factor, which controls adaptation of cancer cells to low oxygen availability (physiological environment for malignant tumor growth) and angiogenesis; BCL-XL, a group of anti-apoptotic proteins.

Conversely to the detrimental role of immune evasion pathways in cancer, overactivation of immune responses leads to multiple autoimmune disorders such as rheumatoid arthritis, psoriasis, multiple sclerosis, affecting a large number of individuals worldwide. Autoreactive T cells are considered as key regulatory and effector cells in these autoimmune diseases (10). Therefore, pharmacological correction of immune checkpoint pathways is desirable for these diseases, and may result in fundamentally novel and highly efficient therapeutic strategies.

The goal of this Research Topic is to provide and summarize recent advances in our understanding of the molecular and cellular mechanisms of anti-tumor immunity and autoimmune disease and, importantly, biochemical regulation and pharmacological correction of these immune networks.

The work by Brauneck et al. highlights co-expression of TIGIT with PD-1, TIM-3 or CD39 in Vδ1 T cells in acute myeloid leukemia (AML) and myeloma. γδ T cells are a unique subpopulation of T cells which recognize cancer cells through T cell receptors but also *via* NK cell receptors and are not dependent on major histocompatibility complex-mediated antigen presentation.

The work by Schlichtner et al. reports the discovery that VISTA expression is regulated by the TGF-β-Smad3 pathway, and highlights that the differential nature of this effect is most likely associated with nuclear compartmentalisation.

The article by Geanes et al. describes the development of combinational antibody therapies for diffuse large B cell lymphomas. This study highlighted that bispecific antibodies, which target multiple B cell receptors expressed by lymphoma cells, potentially deliver improved defense against relapse of the disease and its resistance to immunotherapy.

The paper by Mandour et al. showed for the first time that inhibition of IL-12 heterodimers impairs the protective effect of Toll-like receptor (TLR) 9 stimulation in preventing early mouse plasmacytoma cell growth, highlighting the crucial anti-cancer role for IL-12-mediated innate immunity caused by infections.

The mini review written by Turner et al. discusses the role of CXCR5+CD8 cytotoxic T cells in cancer and autoimmunity, including potential repercussions during immune checkpoint blockade therapy. This review analyses CXCR5+CD8 T cells as potential immunotherapy targets or possible drivers of immune-mediated adverse events.

The mini review by Gurrea-Rubio and Fox highlights the dual role of cluster of differentiation (CD) 6, a type I transmembrane glycoprotein belonging to the highly conserved scavenger receptor cysteine-rich superfamily of proteins, as a target for anti-cancer therapy and treatment of autoimmune disease.

The article by Potashnikova et al. describes the study of cytotoxic T cells from patients with atherosclerosis which exhibit increased adhesiveness and distinct integrin expression patterns.

The paper by Thurau et al. describes a clinical trial where a new small molecule dihydroorotate dehydrogenase (DHODH)-inhibitor [KIO-100 (PP-001)] was used to target activated T cells with the purpose of intraocular treatment of autoimmune uveitis. DHODH is an enzyme catalyzing the fourth enzymatic step, which involves the ubiquinone-mediated oxidation of dihydroorotate to orotate in *de novo* pyrimidine biosynthesis.

The study by Sato et al. discovers that salivary gland conventional NK cells may enhance autoreactive responses in target organs by upregulating IFN-γ production, whereas salivary gland-resident NK cells protect target cells against T cell cytotoxicity.

And the article by Chen et al. describes the clinical factors associated with blood transfusion in lymphedema liposuction surgery, comparing autologous and allogeneic transfusion patterns in lymphedema patients.

In conclusion, this Research Topic includes 5 original research papers, 2 brief research reports, 1 phase I clinical trial study and 2 mini-reviews. The articles highlight the novel aspects of anti-cancer immune evasion machinery and pathological reactions of T cells in cancer and autoimmune disease, supporting the concept of complex biochemical networks regulating cytotoxic immune responses.

## Author contributions

VS, BG, and EF-K wrote the manuscript and created the figure. All authors contributed to the article and approved the submitted version.
